# Mental health needs among patients experiencing extended antepartum hospitalization: A qualitative study

**DOI:** 10.1002/pmf2.70229

**Published:** 2026-01-19

**Authors:** Alison N. Goulding, Myriam Ibarra, Nicole Cirino, Emily S. Miller, Terri L. Fletcher

**Affiliations:** ^1^ Department of Obstetrics and Gynecology Division of Maternal‐Fetal Medicine Baylor College of Medicine Houston Texas USA; ^2^ VA HSR&D Houston Center of Innovations in Quality Effectiveness and Safety Michael E. DeBakey VA Medical Center Houston Texas USA; ^3^ Department of Obstetrics and Gynecology Division of Reproductive Psychiatry Baylor College of Medicine Houston Texas USA; ^4^ Menninger Department of Psychiatry and Behavioral Sciences Baylor College of Medicine Houston Texas USA; ^5^ Department of Obstetrics and Gynecology Division of Maternal‐Fetal Medicine Warren Alpert Medical School of Brown University Providence Rhode Island USA; ^6^ VA South Central Mental Illness Research Education and Clinical Center Houston Texas USA

**Keywords:** antepartum, anxiety, depression, mental health, perinatal, pregnancy, qualitative

## Abstract

**Introduction:**

Hospitalized antepartum patients are at risk for development or exacerbation of a mental health condition, with one in three screening positive for depression or anxiety. High‐quality data are needed to characterize mental health needs and inform evidence‐based interventions in this high‐risk population. This study aimed to assess the perspectives of patients experiencing extended antepartum hospitalization regarding current mental health needs and potential interventions.

**Materials and Methods:**

We interviewed hospitalized antepartum patients at a large referral center from May to September 2024. English‐speaking adults admitted for at least 7 days were eligible. Those with stillbirth, major fetal anomalies or planned fetal surgery, intensive care unit admission, or primary psychiatric concerns were excluded. Individual, semi‐structured interviews were conducted with each participant, including questions on impact of hospitalization on mental health, current stressors, and available support. Interviews were audio‐recorded and transcribed. Rapid qualitative analysis was performed, and overarching themes were identified.

**Results:**

Participants included 23 antepartum patients hospitalized for at least 7 days. Mean gestational age was 26.6 weeks; mean hospitalization duration was 26 days. Common reasons for admission included preterm prelabor rupture of membranes (*n* = 7, 30%) and placenta accreta spectrum (*n* = 5, 22%). Rapid qualitative analysis identified five themes: (1) emotional distress during hospitalization; (2) varying stressors, depending on individual life circumstances; (3) feeling safe in the hospital while missing home; (4) openness to engaging in therapy during hospitalization; and (5) belief that increased interpersonal interactions would improve mental health.

**Conclusions:**

Collectively, patients experiencing extended antepartum hospitalization describe unmet mental health needs and the desire for help navigating stressful life events during hospitalization. Findings from this qualitative study provide important insights into the mental health needs of patients experiencing extended antepartum hospitalization, a unique and high‐risk population. These findings can inform future work to develop novel interventions to support mental health in patients experiencing antepartum hospitalization.

## 1 INTRODUCTION

Perinatal mental health conditions are common, affecting more than one in five women [1, 2]. Depressive and anxiety disorders are the most common conditions [2]. Once diagnosed, many perinatal patients, unfortunately, do not receive any form of mental health treatment [3, 4]. Untreated mental health conditions contribute to adverse maternal outcomes, including worsened chronic conditions, substance use, and increased maternal morbidity and mortality [5, 6]. Mental health conditions are a leading cause of maternal mortality in the United States, contributing to over 20% of pregnancy‐related deaths, all of which are preventable [7, 8]. Untreated perinatal mental health conditions are also associated with adverse obstetric (fetal growth restriction, preterm birth, stillbirth) [6, 9, 10] and child (impaired neurodevelopment and behavior) [11, 12] outcomes, and they are estimated to cost the US $14.2 billion annually [13].

Hospitalized antepartum patients are at increased risk for perinatal mental health conditions, with one in three screening positive for depression or anxiety, twice the prevalence in the general obstetric population [14]. This high‐risk population faces numerous mental health stressors. The experience of pregnancy complications itself imparts increased risk for depression and anxiety symptoms [15]; the stress, social isolation, and sleep disruption that occur during hospitalization exacerbate these risks [16]. Most hospitalized antepartum patients will give birth preterm, and parenting a preterm baby with the associated medical complications can additionally harm mental health [17, 18].

Among low‐risk perinatal populations, qualitative studies have found that psychological processes, including guilt, avoidance, adjustment difficulties, and loneliness, contribute to emotional distress [19, 20]. Other qualitative work has explored barriers to engagement in perinatal depression treatment in the outpatient setting, finding numerous barriers at the levels of the individual, clinician, and society [21]. Hospitalized antepartum patients, often facing unexpected pregnancy complications and separated from usual sources of support, have unique mental health needs. Those admitted for extended periods are anticipated to face more substantial stressors and to be at even higher risk for perinatal mental health conditions. Existing perinatal mental health interventions have been designed for low‐risk outpatients [22, 23, 24, 25], and they are not aligned with the unique needs of hospitalized antepartum patients.

Despite this substantial disease burden, there is limited research on mental health needs in hospitalized antepartum patients. A recent meta‐analysis [26] identified only three trials focused on mindfulness, stress reduction, and psychoeducation. The few existing longitudinal studies report conflicting findings regarding the trajectory of mental health symptoms throughout the antepartum hospital stay [14]. To our knowledge, no prior studies have used qualitative methodology to examine mental health experiences of hospitalized antepartum patients. To fill this gap, this study aimed to assess the perspectives of patients experiencing extended antepartum hospitalization on current mental health needs and preferences for potential interventions.

## 2 MATERIALS AND METHODS

We performed individual, semi‐structured interviews with pregnant patients admitted for an extended period to the unit for hospitalized antepartum patients at a regional referral center serving a large, diverse population with a broad range of high‐risk obstetric and medical conditions. To be eligible, participants were required to be: (1) admitted for 7 days or more; (2) fluent in English; (3) 18 years or older. We elected to include only patients admitted for 7 days or more to select clinically stable patients with ample exposure to the inpatient environment. Participants were excluded if they were admitted for a primary psychiatric concern or if they required admission to the intensive care unit. Other exclusion criteria included stillbirth, major fetal anomalies, or planned fetal surgery. Eligibility was determined through electronic medical record screening. Eligible participants were approached in‐person and offered study enrollment. For those who elected to enroll and provided informed consent, interviews were conducted in‐person by a master's‐level research coordinator (M.I.) with no prior relationship with the participants. No members of the study team were involved in participants’ medical care. Interviews were conducted in the participant's private hospital room. Participants received a gift card as compensation. Interviews were conducted between May and September 2024.

The semi‐structured interview guide (Table 1, File S1) was informed by an in‐depth literature review and guidance from our multidisciplinary team. The interview guide was pilot tested within the study team. Our team is composed of individuals with varying clinical and research expertise. A.G. and E.M. are high‐risk obstetricians with expertise in perinatal mental health. T.F. is a clinical psychologist with expertise using mixed qualitative and quantitative methods to evaluate the implementation of mental health care. N.C. is an experienced reproductive psychiatrist. M.I. (research coordinator) has a master's degree in public health and substantial training and experience in qualitative research. Interview guide domains included the impact of hospitalization on mental health, current stressors, perceptions of support from the community and medical care team, and inpatient resources to support mental health. Interviews lasted an average of 71 min (range: 33–163 min) and were audio‐recorded and professionally transcribed. Transcripts were de‐identified. The sample size was determined based on thematic review and saturation of themes.

**TABLE 1:** Examples of open‐ended questions used in semi‐structured interviews to assess perspectives of hospitalized antepartum patients on current mental health needs and potential interventions.

How would you describe your mental health today? Can you please describe your current stressors? How has this hospital admission impacted your mental health? How does your community (defined as your close family and friends whose opinions you respect) talk about mental health? What does support look like to you? How do the people closest to you support you? Can you please describe what, if anything, you have already shared with your medical team about your mental health? What could your medical team do to improve your mental health? What kinds of activities do you think could help to improve your mental health throughout your hospital stay?

Individual interview transcripts were summarized using rapid qualitative data analytic techniques incorporating both deductive and inductive approaches [27]. Rapid analysis is a systematic way to analyze qualitative data that produces results similar to lengthier qualitative analysis approaches [28, 29]. Following established rapid qualitative analysis methodology [30], the research coordinator (M.I.) and the first author (A.G.) reviewed interview transcripts. Summaries based on interviewer notes were organized deductively by domain (each of which aligned with individual interview questions) into a matrix. M.I. and A.G. reviewed the matrix and generated an initial set of themes with supporting quotations using an inductive approach in which theme development is rooted in the content of the dataset, rather than guided by pre‐existing theories. These themes were refined and finalized through a series of study team meetings including M.I. and A.G., in addition to T.F. (senior author with expertise in rapid qualitative analysis). Differences in interpretation and categorization were discussed at team meetings and resolved through consensus, with T.F. serving as tie‐breaker and final reviewer. Other coauthors served as additional sources of peer review and guidance in the interpretation of results.

This study was approved by the Institutional Review Board. In accordance with this approval, we received and archived written patient consent for each participant. Reporting of qualitative results follows the Consolidated Criteria for Reporting Qualitative Research (COREQ) standards [31].

## 3 RESULTS

**FIGURE 1:**
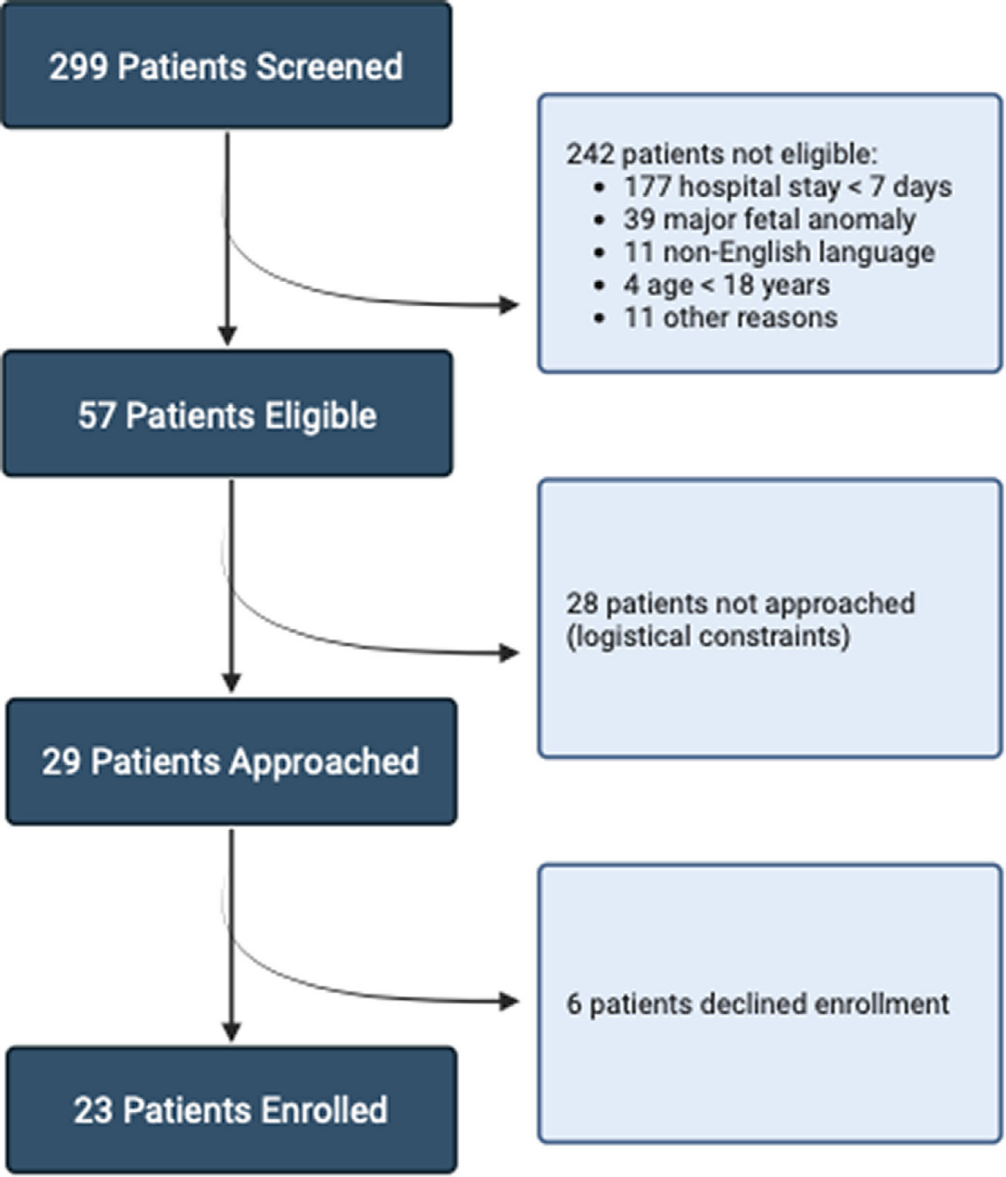
Participant recruitment flowchart. This figure describes the recruitment enrollment process, including numbers of patients screened, eligible, approached, and enrolled, for a final study sample including 23 hospitalized antepartum patients enrolled as participants.

From May to September 2024, 299 patient charts were screened, and 57/299 (19%) were determined eligible for this study. Most patients (177/242, 73%) were ineligible due to antepartum hospital stay duration less than 7 days. See Figure 1 for details regarding recruitment. Twenty‐nine patients were approached for study enrollment; 23 patients provided consent and were enrolled (23/29, 79%). Thematic saturation was achieved after 23 interviews, and enrollment was halted at this time. Mean gestational age on admission was 26.6 weeks (range: 10.4–35.6 weeks), and mean duration of hospital stay was 26 days (range: 7–48 days). The most common reasons for admission included preterm prelabor rupture of membranes (*n* = 7, 30%) and placenta accreta spectrum (*n* = 5, 22%). Mean maternal age on admission was 35 years (range: 21–50 years). Most participants were multiparous (*n* = 20, 87%). Participants were racially and ethnically diverse, self‐identifying as Hispanic White (*n* = 7), non‐Hispanic Black (*n* = 7), non‐Hispanic White (*n* = 7), and Other (*n* = 2). Further descriptive characteristics are presented in Table 2. Rapid qualitative analysis identified five overarching themes, described below. Table 3 provides exemplar quotes for each theme, along with relevant clinical characteristics.

**TABLE 2:** Characteristics of interviewed participants on the antepartum hospital unit (*n* = 23).

Characteristic	*N* (%) or Mean (min–max)
Age (years)	34.5 (21–50)
Race	
Black	7 (30)
White	14 (61)
Other^a^	2 (9)
Hispanic Ethnicity	7 (30)
Insurance	
Private	14 (61)
Public	9 (39)
History of diagnosed mental health conditions	8 (35)
Multiparous	20 (87)

^a^ The two participants identifying as “Other” racial groups included one participant identifying as biracial and another participant who preferred not to provide this information.

**TABLE 3:** Overarching themes expressed by hospitalized antepartum patients in individual interviews (*n* = 23).

Theme	Exemplar quotes	Participant characteristics (maternal age, gestational age, reason for admission, race, and ethnicity)
Emotional distress during hospitalization. Participants expressed emotional distress associated with their current hospitalization.	“I feel like I'm going to die…Some days I feel like I'm going to give up, like whether it be mentally, like my body is going to give up, or I'm mentally going to check out.”	34 years, 23 weeks of gestation, PPROM, non‐Hispanic biracial
	“And I was like, because I can't afford to put anything else in my basket. My basket is full.”	37 years, 21 weeks of gestation, PPROM, Hispanic White
	“Because it's been more of like anxiousness and nerves and, you know, like initially when you hear about this, and they tell you all the risks, and you hear the complications of everything. It's like the initial thought was like well, am I going to be okay? I have a three‐year‐old at home, and like the thought of that was scary…”	36 years, 31 weeks of gestation, PAS, non‐Hispanic White
Varying stressors depending on individual life circumstances. Participants described a wide range of current stressors related to different factors.	“My being here [in the hospital] trying to control the [home] situation is driving me nuts. There is nothing I can do because I'm here; so it's just stuff that's going to get dealt with how ever it gets dealt with, with me not being there. It's frustrating for me.”	36 years, 29 weeks of gestation, PAS, non‐Hispanic Black
	“Just everything…The only thing that's not stressing me out is that I don't have to…go to work, but that's also a stressor because…I'm not making money.”	34 years, 23 weeks of gestation, PPROM, non‐Hispanic biracial
	“My children are starting school and I'm not there."	42 years, 30 weeks of gestation, PAS, non‐Hispanic White
Feeling safe in the hospital while missing home. Participants appreciated the physical safety offered by the hospital environment, while simultaneously missing being at home with their families.	“It's really a lot, and that is where I feel bad because…I need to be here because it's the safest place for me and our daughter. But there's a lot of responsibilities at home. I want to be in two places at once.”	34 years, 23 weeks of gestation, PPROM, non‐Hispanic Black
	“It's hard being here, but I feel like safe being here. Like I know that I am in the best hands here; and if something was to happen, I'm here and they can save the baby and like everything is going to be okay.”	35 years, 31 weeks of gestation, preterm labor, non‐Hispanic White
	“Everyone here is lovely; I like it here and I want to stay here. I know the longer I'm here, the better it's going to be. But also, there is this part in me that like is freaking out, that like I don't know, I kind of feel like I'm in a box a little bit.”	31 years, 22 weeks of gestation, PPROM, Hispanic White
Openness to engaging in therapy during hospitalization. Participants expressed interest in and openness to therapy during their hospitalization.	“It's not a joke when it comes to mental health…So, someone [to lead therapy] who…knows how we feel. Knows oh, this is okay. That's not okay. So, someone who has experience and also could be going through the same thing that the exact same person in that room that they're talking to is going through.”	22 years, 24 weeks of gestation, PPROM, non‐Hispanic Black
	“Yes, because I feel like the more understanding I get, the more wisdom, it helps me to kind of put things into perspective and not let things consume me.”	43 years, 20 weeks, of gestation, cervical insufficiency, non‐Hispanic Black
	“I think it's important…I'm not afraid to say I have a therapist because everything could be perfect, but sometimes you just need somebody to talk to, to vent to…You know we take care of everybody else and sometimes we don't take care of ourselves.”	39 years, 24 weeks of gestation, PPROM, Hispanic White
Belief that more interpersonal interaction would improve mental health. Participants expressed the desire for increased interpersonal interactions and the belief that these interpersonal interactions would improve mental health.	“Support is kind of like what we're doing now. Somebody just sitting. You don't always have to give a solution. Just let me get it out. That's support enough for. I don't need an answer. I don't need a ‘Well. I'm glad you feel better. I'm glad you got that off your chest.’ That's support for me.”	31 years, 28 weeks of gestation, preeclampsia, non‐Hispanic Black
	“Like you came. And I'm, like, okay. Yeah, I'll talk to you. Not that you're a therapist, but I mean, opening up to you about stuff makes me feel better…I'm going through a bunch of struggle!”	34 years, 23 weeks of gestation, PPROM, non‐Hispanic biracial
	“Seeing somebody else. Like having that face‐to‐face conversation really would make a difference.”	23 years, 24 weeks of gestation, PPROM, Hispanic White

Abbreviations: PAS, placenta accreta spectrum; PPROM, preterm prelabor rupture of membranes.

### 3.1 Emotional distress during hospitalization

Many participants described intense anxiety and distress at the start of their hospital stay, as they adjusted to separation from family and friends and processed new risks to their pregnancy. Several participants described recurrent bouts of crying and difficulty coping. One participant described the sense that they were “stuck” and that their life had “fallen apart” since being hospitalized (40 years, 27 weeks of gestation, Hispanic White). Participants described the harmful impacts of these emotions on their overall health, with one participant verbalizing the sense that “my body is going to give up, or I'm going to mentally check out” in the face of this stress (34 years, 23 weeks of gestation, non‐Hispanic biracial).

### 3.2 Varying stressors depending on individual life circumstances

Participants described a wide range of current stressors. Family responsibilities were frequently described as a source of stress, specifically, not being present to complete routine caregiving duties and needing to find alternative childcare arrangements. Many participants described a lack of support, particularly with childcare responsibilities, beyond their nuclear family, making the current hospitalization especially challenging. Participants described sadness at missing their children's milestones, such as starting school, and frustration at things being done differently at home while they were in the hospital. Many described baseline financial stress, now exacerbated by inability to work while hospitalized. Others expressed challenges transitioning professional responsibilities to others while they were unable to work, and concerns about loss of employment caused by prolonged hospitalization and absence from work.

### 3.3 Feeling safe in the hospital while missing home

The experience of hospitalization often elicited conflicting emotions in participants. They voiced appreciation for the physical safety offered by the hospital environment, while simultaneously expressing how much they missed being at home with their families. The balance of these conflicting emotions differed for individual participants. Some reported predominant feelings of safety and relief to be in the secure hospital environment. For these participants, the physical safety of the hospital environment, specifically the proximity to medical providers who could respond rapidly in an emergency, helped them to relax and decreased their overall levels of stress. Other participants reported principally feeling sad to be separated from loved ones. For some participants, these conflicting emotions produced stress and the desire to “be in two places at once” throughout their hospital stay (34 years, 23 weeks of gestation, non‐Hispanic Black).

### 3.4 Openness to engaging in therapy during hospitalization

Most participants expressed openness to and interest in engaging in therapy during their hospitalization. Several framed the hospitalization as a unique opportunity to engage in therapy that patients “may not ever…seek otherwise” and that therapy “may be something that changes their lives” (43 years, 20 weeks of gestation, non‐Hispanic Black). Some participants described positive experiences with therapy before the current hospitalization, and they desired to continue therapy in the hospital. Others had not previously engaged in therapy but expressed the need for additional support through therapy to cope with their current stressors. Participants had different preferences for individual versus group therapy formats, but most expressed preference for in‐person therapy (as opposed to virtual therapy).

### 3.5 Belief that more interpersonal interaction would improve mental health

Participants expressed the desire for increased interpersonal interaction during their hospital stay and the belief that increased opportunities for interpersonal interaction would improve mental health. Some participants expressed feeling bored and isolated and longing for more opportunities for social interaction in general. Others specifically voiced the desire for interpersonal interactions with peers with similar lived experiences who could provide support and guidance. Several patients described how “opening up” during the study interview helped them feel better and voiced desire for additional opportunities to connect with others and share their thoughts and feelings (34 years, 23 weeks of gestation, non‐Hispanic biracial). Most participants were open to receiving support from individuals with a range of backgrounds, including traditional mental health professionals (i.e., psychologists or social workers), other members of the medical team, or peers. The predominant desire voiced by participants was to talk with and receive support from someone understanding their unique stressors.

## 4 DISCUSSION

Interviews inquiring about mental health during extended antepartum hospitalization revealed five main themes: (1) emotional distress during hospitalization; (2) varying stressors, depending on individual life circumstances; (3) feeling safe in the hospital while missing home; (4) openness to engaging in therapy during hospitalization; and (5) belief that increased interpersonal interaction would improve mental health. Findings from this qualitative study provide important insights into the mental health needs of patients experiencing extended antepartum hospitalization, a unique and high‐risk population.

This study adds to the limited body of literature on the needs of hospitalized antepartum patients. One of the few large, population‐based studies, a cohort study of 348,848 patients giving birth in California in 2017, found that almost one third of patients experienced antepartum hospital use, with emergency department visits (30.4%) much more common than antepartum hospitalizations (1.2%) [32]. Patients with *any* antepartum hospital use were more likely to experience maternal morbidity or delivery complications, with those experiencing antepartum hospitalization at the highest risk. Specifically regarding mental health risks, symptoms of depression and anxiety are common; a 2021 meta‐analysis found that one in three hospitalized antepartum patients screened positive for depression or anxiety [14]. Antepartum hospitalization is also associated with increased rates of moderately severe‐to‐severe postpartum depression symptoms [33]. Our study's qualitative methodology adds the patient perspective to more fully understand the increased risks described in prior observational cohort studies. We focus on those experiencing extended antepartum hospitalization (7 days or more) to ensure ample exposure to the inpatient environment. Participants in our study described emotional distress as a common experience during antepartum hospitalization, with most individuals describing a need for additional support to cope with new stressors. To our knowledge, this is the first qualitative study to gather information about patients’ mental health experiences during antepartum hospitalization.

In addition to increasing risk, antepartum hospitalization presents unique opportunities for interventions to support mental health. While the hospital environment offers challenges, including stress, social isolation, and sleep disruption [16], our participants described feeling safe in the hospital and openness to engaging in therapy during hospitalization. Indeed, separation from routine roles and responsibilities presents a unique opportunity to engage in care. The benefits of targeted psychological interventions during hospitalization have been noted in other nonpregnant, inpatient populations [34, 35]. However, existing perinatal mental health interventions have been designed for low‐risk outpatients [22, 23, 24, 25]; they are not aligned with the perspectives and needs of high‐risk inpatients facing acute stressors. For example, the two most widely used programs for preventing perinatal depression, the cognitive behavioral therapy (CBT)‐based Mothers and Babies [22, 23] program and the interpersonal psychotherapy–based Reach Out, Stand Strong, Essentials for New Mothers (ROSE) [24, 25] program, are administered over many weeks and focus on outpatient stressors and transitioning to motherhood in the home setting. Our study adds to the limited existing research on mental health in hospitalized antepartum patients. A recent meta‐analysis identified only three trials focused on mindfulness, stress reduction, and psychoeducation, finding that these interventions modestly but significantly reduced symptoms of anxiety, depression, or stress [26]. Indeed, hospitalized antepartum patients represent a challenging study population due to the heterogeneity of medical conditions and unpredictability of clinical course. These characteristics require tailored interventions responsive to the specific needs of this population. Our study examines essential patient perspectives that must be incorporated to build scalable, sustainable interventions to support mental health in hospitalized antepartum patients.

Our study findings suggest multiple essential characteristics for mental health interventions that meet the needs of hospitalized antepartum patients. Patients describe heterogeneous stressors, including family, finances, employment, and the hospital environment. A transdiagnostic intervention, applicable and responsive to a variety of experiences and needs, is warranted. Potential therapeutic interventions include those based on CBT and problem‐solving therapy (PST). CBT, a proven intervention for both depression and anxiety, aims to reduce symptoms by modifying negative patterns of thinking and behavior [36, 37]. PST aims to enhance well‐being by helping people cope with stressful life problems [38]. PST also has demonstrated efficacy in treating depression [39] and anxiety [40]. Both CBT and PST are responsive to patient‐identified problems and adaptable to the specific stressors of antepartum hospitalization.

Our study findings also suggest that a wide range of individuals, including psychologists, social workers, doulas, community health workers, and peer supporters, could lead mental health interventions for hospitalized antepartum patients. Patients expressed the belief that increased interpersonal interactions in general would improve mental health, and some specifically desired interpersonal interaction with peers with similar lived experiences. Most expressed the overarching desire to receive support from an understanding individual, suggesting that those with a range of backgrounds could be trained to provide this support. Given the severe shortage of mental health professionals across the healthcare system [41, 42], especially for perinatal populations [43], it is critical to identify individuals beyond licensed therapy providers who can lead interventions to improve perinatal mental health. Finally, findings from our study support universally offering mental health interventions to patients experiencing extended antepartum hospitalization. Most patients expressed desire for additional mental health support and were interested in therapy, many more than the one third of patients expected to screen positive on formal screening for depression or anxiety [14]. Broadly inclusive mental health interventions offered to all patients, rather than a “screen‐in” approach, appear best suited to meet the needs of this population.

Study strengths include our diverse, high‐risk antepartum population drawn from a broad region surrounding a referral center providing the highest level of maternal care. Our study employed rigorous qualitative methodology, informed by an experienced and multidisciplinary team, to investigate patient perspectives on the mental health experience during antepartum hospitalization. We had a high response rate (23/29, 79%) when enrolling participants. Finally, no members of the study team were involved in participants’ medical care, reducing the potential for social desirability bias.

Our study also has notable limitations. As is typical with qualitative studies, our sample size was small, which can limit generalizability. Our study enrolled patients within a single hospital center, and findings may not be generalizable to other environments. We specifically did not enroll any non‐English‐speaking participants due to logistical constraints, and patients who lacked insurance coverage were not represented due to the characteristics of our center's patient population. Thus, our findings cannot be generalized to these populations. Additionally, we enrolled only patients admitted for 7 days or more in order to select clinically stable patients with ample exposure to the inpatient environment. These findings may not represent the views of patients admitted for shorter durations, who comprise the majority of hospitalized antepartum patients. Our team felt that perspectives from patients with extended stay durations would be most informative for understanding mental health needs specific to the antepartum hospitalization experience. We also acknowledge the possibility of selection bias, in that participants enrolling in our study may be more open to discussing mental health than the general population. Finally, our study was qualitative in nature and thus aimed to provide a rich description of patients’ perspectives; it was not intended to test specific hypotheses nor to infer causality.

## 5 CONCLUSIONS

Insights from this qualitative study provide detailed information regarding the patient's mental health experience during extended antepartum hospitalization. Participant responses emphasize both the unique stressors and the unique intervention opportunities within antepartum hospitalization. Specific themes included emotional distress during hospitalization, varying stressors depending on individual life circumstances, feeling safe in the hospital while missing home, openness to engaging in therapy during hospitalization, and the belief that increased interpersonal interaction would improve mental health. These findings can inform future work to develop novel interventions to support mental health in hospitalized antepartum patients, a unique and high‐risk population with unmet mental health needs.

## AUTHOR CONTRIBUTIONS

**Alison N. Goulding**: Conceptualization (lead); formal analysis (equal); writing—original draft (lead); writing—review and editing (lead). **Myriam Ibarra**: Investigation (lead); project administration (lead); formal analysis (equal); writing—review and editing (supporting). **Nicole Cirino**: Methodology (supporting); writing—review and editing (supporting). **Emily S. Miller**: Conceptualization (supporting); methodology (supporting); writing—review and editing (supporting). **Terri L. Fletcher**: Conceptualization (supporting); formal analysis (supporting); writing—original draft (supporting); writing—review and editing (supporting).

## CONFLICT OF INTEREST STATEMENT

The authors declare no conflicts of interest.

## ETHICS STATEMENT

This study was approved by the Baylor College of Medicine Institutional Review Board (H‐55242).

## Supporting information



Supporting Information

## Data Availability

The data that support the findings of this study are available from the corresponding author upon reasonable request.
