# Supplementary material for: Mental health needs among patients experiencing extended antepartum hospitalization: A qualitative study
Source: Pregnancy (Hoboken). 2026 Jan 19;2(1):e70229. doi: 10.1002/pmf2.70229 (PMC13344783; doi:10.1002/pmf2.70229)
Supplement: Supplementary file 1 [file PMF2-2-e70229-s001.docx]

**Supplemental File.** Full semi-structured interview guide for manuscript, “Mental health needs among antepartum patients: A qualitative study.”

**Hospitalized Antepartum Patient Interview**

*Thank you for taking the time to speak with me today. This is a study seeking to better understand your mental health and how it is impacted by your pregnancy and specifically by this hospital stay. Different people define mental health in different ways. Other phrases include “mental wellbeing,” “mood,” “emotional health,” and “stress level.” If you prefer to use another term or phrase for “mental health” throughout this interview, please let me know and I am happy to use your preferred language.*

*Our conversation will last about 45-60 minutes. You can stop the interview at any time. You do not have to answer any questions that make you uncomfortable or that you don’t have answers to. Everything you tell me today is confidential. There is no way to tie anything you say directly back to you. We will not share any of this information with your medical team.*

*I would like to record the interview today with your permission. Is that ok with you? If you agree, I’ll start the recorder and then ask again so we can have it on record.*

[If YES, start the recorder and proceed with the following for documentation purposes]:

- *This is [name of interviewer] interviewing participant number [number]. Today is [today’s date].*
- *This recording will be used solely for this research project and will not be shared with anyone outside of the research team.*
- *Do you give verbal consent to participate in the interview for this study and agree to have the interview recorded?*

[If YES, proceed]

[If NO, confirm subject understood the request. If declines to recording, ask if taking written notes during the interview is okay.]

I. *First, I would like to ask a bit about your background and how you are doing during your hospital stay.*

- Can you describe your pregnancy prior to this hospital stay?
  - *Probe:* What about in terms of your physical health?
  - *Probe:* What about in terms of your mental health?
- What brought you into the hospital initially?
- As discussed above, different people define “mental health” in different ways. Other phrases include “mental wellbeing,” “mood,” “emotional health,” and “stress level.” What term do you prefer to use when talking about this topic?

II. *Thank you for sharing that. Next, I would like to ask more specifically about your mental health* *[or substitute preferred language here]*.
1. How would you describe your *mental health* today?
   - How would you describe your *mental health* in general, prior to this pregnancy?
     i. On a scale of 1 to 10, with 10 being optimal mental health and 1 being very poor mental health, how would you rate/score your *mental health* before your current pregnancy?
   - How, if at all, has your *mental health* changed during this pregnancy? How would you describe your overall *mental health* throughout this pregnancy?
     i. On a scale of 1 to 10, 10 being optimal and 1 being very poor, how would you rate your *mental health* right before your hospital admission?
2. Can you please describe your current stressors?

What about distance from family?

What about concerns for your baby?

What about concerns for your health?

What about work?

What about finances?

3. Some patients describe stress related to the uncertainty of their future medical course – are you experiencing this type of stress now? If so, can you please describe this further?

- When you think about your future delivery, how does this impact your *mental health*?
  i. What about your health after delivery?
  ii. What about your baby’s health after delivery?

4. How has this hospital admission impacted your *mental health*?
   - Is anything specifically having a **positive** impact on your *mental health* while you are in the hospital?
     i. *Probe:* For example, some patients describe improved mood after talking to family or friends – do you experience this?
     ii. *Probe:* Some patients describe improved mood through participation in online support groups – do you experience this?
     iii. Do you have any hobbies or activities that boost your mood when you are living at home? Have you been able to incorporate any activities into your hospital stay that boost your mood?
   - Is anything specifically having a **negative** impact on your *mental health* while you are in the hospital?
     i. *Probe*: Are there any aspects of the hospital environment (i.e. noise level, food, unwanted interruptions while trying to rest) that are negatively impacting your mood during your hospital stay?
III. *Thank you for sharing that. Next, I would like to ask more specifically about support from your community and their impact on your *mental health*.*
5. How does your community (defined as your close family and friends whose opinions you respect) talk about *mental health*?
   - How does your community view the act of seeking treatment for *mental health* concerns? Does this view change during pregnancy?
     i. *Probe*: What do people close to you say when the subject of *mental health* is brought up? How has this changed during your pregnancy, if at all?
6. What does support look like to you? How do the people closest to you support you?
   - *Probe*: People can experience emotional support, psychological support, physical support, and/or financial support. What kind of support are you feeling now from the people in your community?
   - Who would you like more support from? What ideally would that support look like?
IV. *Thank you for sharing that with me. I recognize that discussing these topics can be difficult. We are about halfway through the interview – would you like to take a break before continuing? Next, I would like to ask for your input on how your medical team could help support your *mental health*.*
7. Can you please describe what, if anything, you have already shared with your medical team about your *mental health*?
   - How did you feel about sharing this with your medical team? Did you find them to be supportive? Why or why not?

As we begin this next section, I would like to remind you that everything that you tell us is confidential and nothing will be shared with your medical team. If you have specific concerns about your care or important feedback about your care, please notify your nurse, doctor, or any member of your team.

8. What could your medical team do to improve your *mental health*?
   - *Probe*: For example, some people describe how they wish their medical team would pay more attention to their *mental health* during daily interactions. Do you feel this way?
9. Is there anything that your medical team is currently doing that is harmful to your *mental health*? If so, can you please describe that?
10. What kinds of activities do you think could help to improve your *mental health* throughout your hospital stay?
    - What are your thoughts about individual therapy sessions during your hospital stay?
      i. If you think that individual therapy sessions might be helpful, what would you like to focus on in these sessions?
      ii. How often do you think you would benefit from engaging in these individual therapy sessions?
      iii. Who would you like to see leading these individual therapy sessions?
      iv. Would you like these individual therapy sessions to be in-person or virtual?
    - What are your thoughts about group therapy sessions during your hospital stay?
      i. If you think that group therapy sessions might be helpful, what topics do you think they should cover?
      ii. How often do you think you would benefit from engaging in these group therapy sessions?
      iii. Who would you like to see leading these group therapy sessions?
      iv. Would you like these group therapy sessions to be in-person or virtual?
    - What are your thoughts about reading a book or pamphlet to learn new ways to improve your *mental health* during your hospital stay?
      i. If you think that this might be helpful, would you like to follow up with a member of your care team afterwards to discuss what you’ve learned and how it might apply to your life?
    - What are your thoughts about listening to an audio-book or podcast to learn new ways to improve your *mental health* during your hospital stay?
      i. If you think that this might be helpful, would you like to follow up with a member of your care team afterwards to discuss what you’ve learned and how it might apply to your life?
    - What are your thoughts about a self-paced online course or mobile phone app to learn new ways to improve your *mental health* during your hospital stay?
      i. If you think that this might be helpful, would you like to follow up with a member of your care team afterwards to discuss what you’ve learned and how it might apply to your life?
    - What are your thoughts about wellness or self-care activities designed to improve your *mental health* during your hospital stay?
      i. *Probe:* Examples of wellness or self-care activities include meditation, yoga, and massage. What does self-care mean to you?
    - What are your thoughts about an educational activity designed to share specific information about future medical events, such as your delivery or your baby’s medical care after delivery?
      i. How do you think that this type of information would influence your stress level and your mood?
      ii. Do you have any concerns that receiving this type of information could actually increase your stress level?
    - *Virtual reality uses computer modeling and simulation to immerse the user in a computer-generated environment that simulates reality through the use of interactive devices, such as headsets or goggles.*
      i. Have you ever used virtual reality technology before? If so, what was your experience?
      ii. What are your thoughts about using virtual reality technology to improve your mood during your hospital stay?
      iii. What are your thoughts about using virtual reality technology to receive therapy designed to improve your mood during your hospital stay?
11. Is there anything else you would like to share with me that I haven’t asked about?

*Thank you so much. We appreciate everything you have shared with us. This concludes the interview.*
